# Supplementary material for: Nutrition Strategies to Promote Sleep in Elite Athletes: A Scoping Review
Source: Sports (Basel). 2025 Oct 2;13(10):342. doi: 10.3390/sports13100342 (PMC12567717; doi:10.3390/sports13100342)
Supplement: Supplementary file 1 [file sports-13-00342-s001.zip › Supplementary Material (S2) Search Strategy Across Databases.pdf]

Database	Search Items				Additional Limits applied
	Population	Search Terms	Comparison	Outcome	
PubMed	Limited to human studies (elite athletes)	("Nutrition"[MeSH Terms] OR nutrition OR "dietary intake" OR diet) AND ("Sleep"[MeSH Terms] OR sleep OR "sleep quality" OR "sleep duration") AND ("Athletes"[MeSH Terms] OR "elite athletes" OR "professional athletes")			Studies published in English
ScienceDirect	Limited to human studies (elite athletes)	(nutrition OR "dietary intake" OR diet) AND (sleep OR "sleep quality" OR "sleep duration") AND ("elite athletes" OR "professional athletes")			Studies published in English
Google Scholar	Limited to human studies (elite athletes)	"nutrition" AND "sleep" AND ("elite athlete" OR "professional athlete") -review -systematic -meta-analysis			Studies published in English
SPORTDiscus	Limited to human studies (elite athletes)	“nutrition” OR "dietary intake" OR diet) AND “sleep” OR "sleep quality" OR "sleep duration") AND "elite athletes" OR "professional athletes")			Studies published in English
Scopus	Limited to human studies (elite athletes)	(nutrition OR "dietary intake" OR diet) AND (sleep OR "sleep quality"			Studies published in English

		OR "sleep duration") AND ("elite athletes" OR "professional athletes")			
