# Supplementary material for: Nutrition Strategies to Promote Sleep in Elite Athletes: A Scoping Review
Source: Sports (Basel). 2025 Oct 2;13(10):342. doi: 10.3390/sports13100342 (PMC12567717; doi:10.3390/sports13100342)
Supplement: Supplementary file 1 [file sports-13-00342-s001.zip › Supplementary Material (S4) Outcomes of the Paper to Podium Matrix using the included studies.pdf]

Outcomes of the Paper to Podium Matrix using the included studies

Study	Research Context	Research Participants	Research Design	Dietary & Exercise Controls	Validity & Reliability	Data Analytics	Feasibility of Application	Risk/Reward	Timing of Intervention	Total Score
Ferguson et al., 2021	+1: Human participants applied performance outcomes measured.	+2: Elite male Australian Rules players, appropriate population.	+2: RCT design, placebo-controlled, within-subjects, appropriate sample.	+2: Pre-sleep nutrition intake monitored; exercise controlled.	+1: Validated sleep measurement tools and exercise test used.	+1: Statistical significance tested, effect sizes reported.	+2: Whey protein is cheap and easy to apply pre-sleep.	+1: Minimal risk from whey protein, a common dietary supplement.	+2: Pre-sleep timing matches peak intervention window.	14
Greenwalt et al., 2023	+1: Applied setting, subjective recovery/sleep outcomes.	+2: NCAA D1 female soccer players, highly relevant.	+2: Randomised trial with three conditions.	+1: Recovery and diet monitored with logs.	+1: PSQI and RESTQ used, validated tools.	+1: Stats and interpretation are appropriate.	+2: Protein drinks are simple and scalable.	+1: Low risk with high potential benefit.	+2: Pre-sleep strategy tested in-season.	13
MacInnis et al., 2020	+1: Focused on α-lactalbumin and sleep/performance in cyclists.	+2: Trained cyclists in appropriate age group.	+2: Double-blind placebo-controlled crossover trial.	+2: Pre-trial diet and training standardised.	+1: Validated tools used; exercise protocol appropriate.	+1: Appropriate statistics and significance reported.	+1: α-lactalbumin is available and practical.	+1: Low risk from dairy protein supplementation.	+2: Pre-sleep dosing fits the mechanistic rationale.	13
Valenzuela et al., 2023	+1: Human trial on cyclists, measuring functional performance.	+2: Professional cyclists, highly relevant sample.	+2: Three-arm RCT with strong design and blinding.	+1: Controlled intake during camp, but some self-reporting.	+1: Validated measures, familiarisation reported.	+1: Effect sizes and stats provided.	+2: Casein supplementation is feasible and common.	+1: Low risk intervention, with potential performance benefit.	+2: Evening feeding aligns with recovery and sleep physiology.	13

Chung et al., 2022	+1: Human study with elite athletes, focused on applied recovery outcomes.	+2: Elite female hockey players, well matched and described.	+2: RCT design with placebo control, strong protocol.	+1: Training loads and nutrition were monitored during the study.	+1: Validated questionnaires and familiarisation used.	+1: Proper statistical tests with effect sizes.	+1: Tart cherry juice is accessible and manageable.	+1: No side effects, antioxidant-rich, safe.	+2: Administered post-exercise, appropriate for the recovery window.	12
Gratwicke et al., 2023	+1: Human field study with real-world performance implications.	+2: Female rugby players, competitive athletes.	+2: Double-blind, placebo-controlled design.	+1: Diet prior to supplementation tracked loosely.	+1: Sleep assessed via actigraphy and diaries.	+1: GLMM stats with interaction terms used.	+1: α-lactalbumin in powder form is easy to use.	+1: Common dairy protein with no risk.	+2: Evening dosing pre-sleep during training.	12
Harnett et al., 2021	+1: Human study on sleep and soreness in rugby athletes.	+2: Elite rugby players, training status described.	+2: Placebo-controlled trial with repeated measures.	+1: Diet not tightly controlled, but training monitored.	+1: Valid tools and consistent protocol used.	+1: Group comparisons and effects presented.	+1: Probiotic supplementation is feasible.	+1: Low risk, generally recognised as safe.	+2: Administered daily during training blocks.	12
Doherty et al., 2023	+1: Human applied study on food-first strategy.	+2: National-level elite athletes [sailing, athletics].	+1: Open-label, no control group.	0: Standard diet advised but not controlled.	+1: Multiple validated subjective tools used.	+1: Descriptive stats and appropriate comparisons.	+1: Kiwifruit is food-first and feasible.	+2: No adverse events, low-risk whole food.	+2: Dosing ~1 hr pre-sleep, ideal timing.	11
Ergolu et al., 2024	+1: Pilot observational study, relevant population.	+2: Elite athletes described by age and sport.	+1: Pilot, no control group, small sample.	+1: Dietary patterns surveyed, not controlled.	+1: Survey tools used, though not all validated.	+1: Exploratory statistics provided.	+1: Observational, no direct intervention.	+1: No risk; dietary observation only.	+2: Explores habitual intake and effects.	11
Condo et al., 2022	+1: Observational human study linking diet to sleep.	+2: Elite female athletes across sports, well described.	0: Observational, no intervention or control.	+1: Dietary intake logged but not controlled by researchers.	+1: Validated nutrition and sleep tools used.	+1: Regression and appropriate analytical approaches.	+1: No intervention, observational only.	+1: No intervention, hence, no risk.	+2: Real-life intake and timing patterns assessed.	10

Falkenberg et al., 2021	+1: Human athletes, but observational, no intervention.	+2: Elite male AFL players well characterised.	0: Observational design limits causal inference.	+1: Dietary intake logged, but not researcher controlled.	+1: Validated survey tools used.	+1: Appropriate analyses reported.	+1: Observational data applicable to athletes.	+1: No intervention, thus no risk.	+2: Examines real-world timing and intake habits.	10
Yasuda et al., 2019	+1: Cross-sectional study on dairy and sleep in elite athletes.	+2: Japanese national athletes, age and training matched.	0: Observational, no control or randomisation.	+1: FFQ data, but self-reported only.	+1: Sleep quality survey tool validated.	+1: Logistic regression used.	+1: Dairy intake practical and low cost.	+1: Dairy is low risk and well tolerated.	+2: Frequency/timing of intake assessed via FFQ.	10
